# The Primary Physiological Purpose of the Membrana Tympani*Read in the Otological Section of the American Congress of Physicians and Surgeons, Washington, September, 1888.

**Published:** 1888-10

**Authors:** S. O. Richey

**Affiliations:** Washington, D. C.


### THE PRIMARY PHYSIOLOGICAL PURPOSE OF THE MEMBRANA TYMPANI.*

BY S. O. RICHEY, M. D.
Washington, D. C.

In the development of anatomical structure, special organs, or parts of organs, come into existence as change of environment, or preparation for change of environment, demands them, for the perpetuation of individual existence. Aquatic animals have some peculiarity in various organs which fits them for the medium in which they live, but these organs are *un*fitted for life in the air. Land animals by certain other peculiarities of structure are *un*fitted for existence under water. Amphibia, by organic structure and therefore organic function, are qualified for both varieties of life. The young of amphibians, spawned in water, have branchiæ, the breathing apparatus of fish, but, with one or two exceptions, adults have lungs instead, the branchiæ giving origin to other needful appendages. Fishes, living always in the water, have no eyelids. Many of them have movable folds, initial upper and lower lids, while in some sharks there is a third fold, forming a nictitating membrane. Most commonly the integument continues immediately into the cornea. In reptiles and birds there is an upper and a lower movable eyelid *and* a nictitating membrane. In some lizards and in serpents, the eyelids are an annular fold, which becomes a pellucid, or transparent, membrane in front of the eye *protecting the eye from the external medium* (Gegenbaur, Comparative Anat-

* Read in the Otological Section of the American Congress of Physicians and Surgeons, Washington, September, 1888.

omy, p. 532). The lachrymal gland appears first in amphibia and reptiles. In reptiles, birds, and some mammals the Harderian gland, the secretion of which is not exactly like that of the lachrymal gland, opens under the nictitating membrane, or the pellucid continuation of integument. These glands are alike in reptiles and birds. In amphibia, even the efferent duct is foreshadowed by an epithelial development, forming a canal to carry off the secretions of the gland.*

The first rudiment of an internal ear is an involution of the integument into a small sac, and occurs among the lower fishes. The crayfish has a simple auditory arrangement, consisting of an auditory sac, or involution of the integument, with fluid contents, a nerve, whose filaments enter auditory hairs, which project into the fluid contents of the sac; no middle or external ear exists. The lamprey possesses two semi-circular canals, and a sacculated vestibule as an auditory organ. Crocodilia have external ear openings closed by a flap of the integument, and this is the earliest suggestion of an outer or drum membrane which I can find in the history of the development of animal life. The ears of birds resemble those of crocodiles. All amphibia have a fenestra ovalis with a cartilaginous, or osseous, columelliform stapes, the expanded proximal end of which is fixed to the membrane of the fenestra (Huxley). Many, if not all, batrachia possess a fenestra rotunda; some have no tympanic cavity or membrane, though tympanic cavities exist in others, communicating with the throat, and tympanic membranes exist to which the stapes is attached. The tympanic membrane in the aglossa is a cartilaginous expansion of the columella. Except the frog, most amphibia have no tympanic membrane. The fenestra *ovalis* only may be found, which is connected with the exteriorly situated membrana tympani in the frog by three auditory ossicula. In reptiles and birds there is one columella. The auditory structures are alike in the lowest mammals and birds; they are similar in man and the higher mammals.

To sum up, the eye, the breathing apparatus, and the ear are more superficially placed in the lower forms of animal life, inhabiting water. As they approach higher forms of life and change their environment they adopt certain changes in structure better adapted to their exposure. The eye is protected, in some instances, from the desiccating influence of the atmosphere by a continuation of the integument, that part of which covering the eye becoming transparent; a tear-gland is developed, supplying the eye with moisture under this covering. As this structure varies to that of two lids, which, by separating, increase the exposure of the eye, the secretion of tears becomes more than ever before necessary, and the gland becomes more highly developed, to supply the more rapid evaporation of moisture. The nictitating membrane in the amphibia answers the double purpose of protection from the irritating effect of impure water upon eyes accustomed to the lighter medium—air; and from the irritation arising from the rapid evaporation caused by the air.

The breathing apparatus of aquatic animals, the branchiæ, being superficially placed, are, for the same reasons, unsuited to amphibious and land animals. The lungs are centrally situated; air, to reach them, must pass over a considerable extent of moist membrane, and is thus materially modified. Is it not most rational that for a similar purpose the auditory apparatus is fixed farther from the surface, and similarly protected? The denizen of

* Naturally here arises a question in regard to the heredity of the *hyperopic* eye, which is the common form in the young of the human race. In water, the refractive power of which is greater than of air, the refraction of the eye needs to be less than in the human being. This suggestion is fairly indicated by a comparison between the states of refraction of the eyes in savages and in civilized races; in the refraction of the young and the adults of civilized races; and in the tendency to *increase* of refraction by heredity and other causes among the more highly civilzed, and hence older races.

the sea needs no conducting apparatus better than the element in which he moves, as the power of conduction of sound, inherent in water, is less only than that of solids. Hence, an auditory sac, for the perception of sound, is all that is necessary, and we find him so supplied. The sac is kept in a condition of proper pliability by the water.

This leads us to a purpose of this paper —the important function of the membrana tympani in man.

The delicacy and pliability of the membrana secondaria must be preserved. It is therefore deeply situated, the external air reaching it only after passing over the mucous lining of the nares, pharynx, and Eustachian tubes, and becoming so saturated with moisture and modified in temperature, as not to subtract moisture from the delicate tissues of the auditory apparatus and lessen their pliability. It is the chief purpose of the membrana tympani to protect the tympanic cavity from the access of unmodified air in its direction. The whole structure of the conducting apparatus subservient to the delicacy and pliability of the membrana secondaria, at the same time aiding, and not repressing, the effect of the sound-waves upon the labyrinthine fluid. A perforation of the membrana tympani little, if at all, affects *immediately* the function of the organ. In time, direct exposure to the air causes gradual change in the structures of the cavity, and therefore impaired function. The smaller the perforation the more gradual the influence, but *any* opening must be similar in its effect upon the structures of the cavity to that of an opening into any other joint, for this is, to all intents, a large joint. In Stricker's Physiology, p. 969, we read: "The articulating surfaces of the ossicula resemble other true joints—they have capsules and a layer of hyaline cartilage on the articulating surface." * * * "The ossicula in adults are covered by mucous membrane, and a very thin periosteum." Besides, there are other tissues in this cavity to whose proper function moisture is necessary, and to which the action of unmodified air must prove deleterious—muscles and membranes. Though the articulations of the ossicula must be impaired in their movements by the chilling and desiccating effects of the air reaching them so directly, this would not express itself in impaired hearing, so long as the membrana secondaria is not stiffened, or excluded from the direct impact of sound-waves by closure of the perforation in the membrana tympani. What otologist has not seen cases in which, with an apparently normal condition of the middle-ear structures, decided increase of impaired hearing would follow a cicatricial closure of such perforation, to improve when sufficient time had elapsed to enable the tissues to become pliable from the natural secretion of the lining membrane? When the lining membrane is disabled by disease from producing this necessary moisture, the impairment of hearing is likely to persist. We will do well to remember that the tympanic cavity is much in the nature of a *serous* cavity, subject to the same maladies as a joint deprived of its capsula, when the drum membrane is removed by disease or otherwise, or when the cavity is exposed by a persistent perforation of the membrane, though not quite so intolerant as a joint. In amphibia the labyrinth is relatively smaller than in fishes; smallest in the mammalia. Thus the nearer to full protection from the direct action of the air, the more developed the tympanic membrane and the deeper seated the labyrinth, the smaller it is, and the less *fluid* it will contain. What is the relation between this fact and the processes of exosmosis from the labyrinth of the fluid contained by it, and evaporation of this fluid? In oto-sclerosis, or when perforation of the membrane exists, may not profound, almost total, deafness be due, in part, to exosmosis and evaporation of the fluid contents of the labyrinth, through an Eustachian tube of enlarged calibre, limiting motion of the perceptive elements by diminution of the fluid in which they float, as well as by anchylosis of the ossicula, or other morbid changes of the middle-ear structures?

We find in Gegenbauer's Comparative Anatomy (p. 527), after his discussion of the labyrinth: "Other parts are gradually added as accessory organs to the auditory organ, although primarily having no relation to it." By "accessory organs" he indicates the development of the bony tympanic cavity from the first branchial cleft; the involution of the pharyngeal mucous membrane forming the Eustachian tube; the involution of the integument, forming the external meatus; the formation, at the points of overlapping of these canals, of the drum membrane; these two tissues, with the *membrana propria*, *developed from the connective tissue lying between*,* making up the tympanic membrane.

Evidently the development of these structures *subsequent* to the existence of the perceptive organ, and *exterior* to it, was with a purpose other than sport. When the simplicity with which nature usually works is borne in mind, we can not believe it was for the purpose of merely making the organ more complex. Can we be certain that the existence of a transmitting mechanism improves the perception? A temporary opening of the tympanic membrane, without other tympanic structural change, does not noticeably impair hearing.

In view of the otherwise guarded position of the labyrinth, is it not most rational to suppose the tympanic membrane to exist, also, for that purpose; especially, with the history of the contemporary development of the eye and its appendages, and the lungs with their more central location and appendages, and the protection which this implies?

This theory of the primary purpose of the tympanic membrane opens up a vast field of aural therapeutics for consideration. In reading aural therapeutics, one must be often impressed with the frequency of ad captandum methods, and the sad experiences and failures in manipulating this obscure organ. We see the immediate effect of the air douche; what is the remote result of its frequent repetition? What was the rationale of the endeavor to maintain an opening in the tympanic membrane in "progressive deafness," when by proper management the Eustachian tube could be kept patulous? Why has so much attention been exhausted upon the tympanic membrane, when its changed appearance is so manifestly due to alterations in the structures behind it? The philosophy of surgical violence to this delicate organ, unless in case of destructive disease, is questionable. Nature is wise, and her effort, so persistent, to heal a drum membrane, whether a perforation is the result of surgical violence or of disease, would indicate some other object in the existence of the membrane than the transmission of sound-waves. If this were not so, why should an opening in the membrane, designed by the surgeon, close, regardless of attempts by tents, eyelets, etc., to keep it open?

My impressions of the advisability of an imperforate membrane are so strong that I have never made an opening in it, except to evacuate fluid from the cavity. On the other hand, for more than ten years it has been my systematic practice to endeavor to close such openings. In case of chronic suppuration of the cavity, if the drum membrane is not restored, the discharge will recur at intervals. In no case in which the drum membrane has been restored under my observation have I learned of a recurrence of the suppuration. Such a result pre-supposes the destruction of the pyogenic membrane and all germinal matter, but it also suggests some other purpose for the existence of the tympanic membrane than its participation in the transmission of sound-waves to the internal ear.

* Hunt, Trans. Internat. Otol. Cong., 1876, pp. 104-112.

CONCLUSIONS.

(1) The primary purpose of the tympanic membrane is that of protection of the tympanic cavity from the evaporating influence of the air; to prevent the *parching* and *stiffening* of the membrana secondaria, the joints of the ossicula, the tendons of the tympanic muscles, and to prevent loss of the labyrinthian fluid by evaporation.

(2) It, being necessary for the protection of the cavity, is modified into part of a transmitting mechanism by the existence of the ossicula and their attachments—a secondary physiological purpose.

(3) A permanent opening of the membrane may, under given circumstances, temporarily improve the function of hearing, but is harmful to the ultimate condition of the organ.

(4) The tendency of the membrane to heal is an effort on the part of nature to preserve the function of hearing from the effects of disease or ill-advised surgical interference.

